# Association Between Wisdom and Psychotic-Like Experiences in the General Population: A Cross-Sectional Study

**DOI:** 10.3389/fpsyt.2022.814242

**Published:** 2022-04-18

**Authors:** Zhipeng Wu, Zhengqian Jiang, Zhipeng Wang, Yuqiao Ji, Feiwen Wang, Brendan Ross, Xiaoqi Sun, Zhening Liu, Yicheng Long

**Affiliations:** ^1^Department of Psychiatry, The Second Xiangya Hospital, Central South University, Changsha, China; ^2^National Clinical Research Center for Mental Disorders, Changsha, China; ^3^Xiangya School of Medicine, Central South University, Changsha, China; ^4^Faculty of Medicine, McGill University, Montreal, QC, Canada; ^5^Department of Psychology, Cognition and Human Behavior Key Laboratory of Hunan Province, Hunan Normal University, Changsha, China

**Keywords:** wisdom, psychotic-like experiences, mental health, psychosis prevention, epidemiology

## Abstract

**Introduction:**

Wisdom has been empirically researched as a complex psychological characteristic that is associated with many mental health outcomes. However, its association with psychotic-like experiences (PLEs) remains unclear. This is the first work to assess wisdom, explore its association with PLEs, and test its moderating effect on the relation between the frequency of PLEs and their associated distress in the general population.

**Methods:**

From January 29th to February 5th, 2021, our online self-administered survey recruited 927 participants (ages 14 to 65) from thirteen Chinese provinces. Convenience sampling was employed. We measured wisdom with the 12-item three-dimensional wisdom scale (3D-WS-12) and PLEs with the 15-item positive subscale of the Community Assessment of Psychic Experiences (CAPE-P15).

**Results:**

Using the cut-off value of 1.47 in the mean frequency score, we divided our participants into high-PLEs group (188, 22.1%) and low-PLEs group (663, 77.9%). Three-dimensional wisdom score was decreased in the high-PLEs group compared to the low-PLEs group (Kruskal-Wallis *t* = 59.9, *p* < 0.001). Wisdom was associated with less frequent PLEs (Spearman’s rho = −0.21, *p* < 0.01) and lower distress related to PLEs (Spearman’s rho = −0.28) in the high-PLEs group (all above *p* < 0.001), which were replicated in the low-PLEs group. Notably, wisdom significantly attenuated the distress associated with PLEs [coefficient = −0.018, Bootstrap 95% CI (−0.029, −0.008)], but only in the low-PLEs group.

**Conclusion:**

Our results implicated that wisdom could protect individuals from distressful subclinical psychotic symptoms and wiser individuals have better general mental health.

## Introduction

Wisdom, a complex and trait-based psychological construct that has been consistently discussed in religious and philosophical texts for centuries, has emerged as a new field of empirical research since the 1970s (1). The essence of wisdom research lies in the definition of wisdom and its empirical measurement. Baltes and Staudinger posited that wisdom was embedded in extensive pragmatic knowledge, concentrating on the cognitive and intellectual aspect (2). Clayton supplemented reflective and affective domains to the components of wisdom (3). Sternberg postulated that wisdom derived from an employment of knowledge regarding to the balance of personal and societal interests (4). Josefsson and colleagues’ research on wisdom stressed some aspects related to wellbeing (5). Ardelt developed a three-dimension model to assess wisdom from cognitive (a deep understanding of human existence and relationships), reflective (accurate/unbiased introspection and perspective-taking), and affective (an emotional affinity for others) domains (6). Wang and Chen claimed that wisdom is a comprehensive psychological construct integrating intelligence and morality (7). Although wisdom might be perceived differently in western and eastern cultures, Jeste and Vahia found that the ancient eastern wisdom components are largely in line with modern view of western wisdom, such as emotion regulation and insight (8).

The assessment of wisdom can be based on interviews by experts, such as the Berlin Wisdom Paradigm (9), or self-reported questionnaires, such as the well-recognized 30-item Self-Assessed Wisdom Scale (SAWS) measuring wisdom from five factors: critical life experiences, emotions, reminiscence, openness, and humor; the 39-item Three-Dimensional Wisdom Scale (3D-WS-39) based on the three-dimensional wisdom model (6, 10); and the newly emerged 28-item San Diego Wisdom Scale (SD-WISE) consisting of six domains: social advising, emotional regulation, pro-social behaviors, insight, tolerance for divergent values, and decisiveness (11). It should be noted that self-reported wisdom is highly related to interview-based wisdom and thus suggests an applicability of using self-reported measures in large-scale studies (1). However, these self-administered questionnaires are lengthy and thus limited in use for multifactorial assessments in psychiatric epidemiology. Hence, Thomas et al. developed a 12-item abbreviated Three-Dimensional Wisdom scale (3D-WS-12) that can be administered quickly within the context of epidemiological surveys and show considerable efficiency and validity (12).

A growing body of studies have found that wisdom is associated with multiple health-related outcomes, encompassing better general physical and mental health (13), wellbeing (14), happiness (15), life satisfaction, successful aging, and resilience (11, 16), as well as lower degree of loneliness (17) in the general population. However, the psychiatric assessments involved in these studies mainly covered subclinical anxiety, depressive symptoms, or common positive domains including wellbeing and resilience, while missing the inclusion of psychotic phenomena, a spectrum of less prevalent but even more debilitating subclinical symptoms. Thus, studies covering psychotic phenomena are warranted to get a more comprehensive picture of the association between wisdom and mental health at subclinical level.

Psychotic-like experiences (PLEs), usually known as subclinical delusions or hallucinations, are common in the general population (18–21). In line with the psychosis continuum model, healthy individuals reporting these symptoms are considered to represent a non-clinical psychosis phenotype and thus supposed to be at high risk of schizophrenia-spectrum disorder (20, 22, 23). More recently, under the notion that PLEs usually coexist with affective symptoms including depression and anxiety (24–27), it is also suggested that PLEs are more likely to serve as an indicator of common mental distress, instead of a single risk state for psychosis (28). For both perspectives, psychotic phenomena such as PLEs are recommended to be included as a significant part of assessment in psychiatric epidemiology.

In addition to depressive and anxiety symptoms, previous studies demonstrated that individuals with PLEs, especially delusional experiences, are prone to cognitive bias, for instance, jump to conclusion (JTC), belief inflexibility (BIB) and aberrant salience bias (ASB) (29–31), in line with well-established findings in patients with full-blown psychosis (32–35). Moreover, it was found that patients at ultra-high risk for psychosis showed significant impairments in social cognition that involves the cognitive processes of perceiving, interpreting, and processing social information (36). Further, adults with PLEs presented dysfunction in emotion regulation (ER), which can be regarded as an element of wisdom; and ER is associated with the frequency and distress level of their PLEs (37). However, these studies are not comprehensive since they did not target wisdom as a whole and thus limited in shedding light on the relation between wisdom and subclinical psychotic symptoms. Further studies investigating wisdom as a unitary psychological construct in the population with PLEs are in pressing need because wisdom could protect high-risk individuals for full-onset psychosis.

To the best of our knowledge, this is the first study to investigate the association between wisdom and PLEs and test its moderating role in the relation between frequency of PLEs and their associated distress in the general population. Specifically, adopting the three-dimensional wisdom model, we tested the following hypotheses: (1) wisdom level is decreased in the population with high-level PLEs compared with those having less PLEs; (2) wisdom is negatively associated with the frequency of PLEs and their associated distress; and (3) wisdom attenuates the relation between frequency of PLEs and their associated distress. We expected to see that although wisdom might decrease with the existence of PLEs, wiser individuals may have less frequent and lower distress related to PLEs. Hence, wisdom might be a good intervention target for lowering the social burden of psychosis in the future.

## Materials and Methods

### Participants

This study was designed as an online cross-sectional survey. We adopted a self-reported questionnaire link at the largest online survey platform in China called “Questionnaire Star^1^”. Our online-based data collection was performed between January 29th and February 5th, 2021. A set of self-rating assessments were distributed to students, corporate staff and retired population from 13 different provinces in China (Hunan, Shanghai, Shanxi, Heilongjiang, Jiangsu, Henan, Guangdong, Shandong, Yunnan, Chongqing, Shaanxi, Anhui, and Jiangxi provinces) using convenience sampling method (38). Specifically, we employed thirteen volunteers, who were college students from above-mentioned provinces, to disseminate the questionnaire link and each volunteer reached at least 100 individuals through WeChat (the largest social media application in China) message. Each participant was electronically informed about the purpose of our study and asked to complete an electronic consent inform, and consent from guardians was also requested for those below 18 years old. With their consent, participants completed all the questionnaires before uploading their answers. Each participant who completed our survey was reimbursed five RMB for their time. On average, it took 10 min for each participant to finish these questionnaires.

Our participants were recruited using the following criteria. Inclusion criteria of the study included: (i) able to understand and complete these questionnaires using electronic devices, (ii) consented to participate in this study, and (iii) without history of psychiatric diagnosis. The study was approved by the Ethics Committees of the Second Xiangya Hospital of Central South University.

### Assessments

#### Wisdom

The abbreviated Three-Dimensional Wisdom Scale (3D-WS-12) was refined from the original three-dimensional wisdom scale (3D-WS-39) (6, 12). It contains 12 items covering affective, reflective, and cognitive dimensions of wisdom, and each domain contains 4 items. Answers to each item include range from 1 = strongly agree or definitely true of myself to 5 = strongly disagree or not true of myself. This wisdom screening tool has been translated into Chinese and shown to be valid to assess wisdom in Chinese population (39). Cronbach’s alpha for the total score of 3D-WS-12 in our sample was 0.79, which were 0.71, 0.75, 0.72 for cognitive, reflective, and affective subscales, respectively.

#### Psychotic-Like Experiences (PLEs)

The 15-item positive subscale of the Community Assessment of Psychic Experiences (CAPE-P15) contains two dimensions: frequency and associated distress of PLEs. Each subscale measures the frequency or associated distress of PLEs from three positive psychotic domains: persecutory ideation (PI, 5 items), bizarre experiences (BE, 7 items), and perceptual abnormalities (PA, 3 items) (40). The psychometric properties of the Chinese version of CAPE-P15 were validated (41). Participants completed the questionnaire based on their past 3 months’ experiences. Answers in the frequency subscale include: 1-never, 2-sometimes, 3-often, and 4-nearly always. Answers in the distress subscale include: 1-not distressed, 2-a bit distressed, 3-quite distressed, and 4-very distressed. The total frequency score was divided by the number of valid answers to reach an average frequency score for each participant. An average frequency score of 1.47 was adopted as the cut-off value for participants with high-level PLEs (high-PLEs group) versus participants with low-PLEs (41, 42). In our sample, Cronbach’s alpha for the total frequency score was 0.88, which was 0.87 for the total distress score.

#### Demographic Information, Previous Psychiatric Diagnosis and Substance Use

We obtained the following demographic information from participants: age, sex, education years, and personal history of psychiatric illness. We screened participants’ personal history of psychiatric diagnoses with the question: “Have you ever been diagnosed with any mental disorder?”.

### Data Analysis

Before statistical analysis, we excluded participants with any previous psychiatric diagnosis to focus on studying subclinical symptoms. We also eliminated subjects whose age apparently did not match their education years and those who spent very little time (less than 3 min in total) on the survey, to filter out those potentially flawed samples.

Our analysis included three major steps. First, we examined the normality and skewness of all measurements using the Shapiro–Wilk test. Since these variables did not meet the assumption of normality (*p* < 0.05), we employed non-parametric methods in the following analysis. Then descriptive analysis was used to present the characteristics of our sample. Second, we divided the participants into two groups (the high-PLEs and the low-PLEs group) using the cut-off value of 1.47 in the mean frequency score of CAPE-P15 (41, 42) and compared the group difference in all variables using Chi-squared test or Kruskal-Wallis test. Then, Spearman correlation was employed to investigate the association between wisdom and frequency and associated distress of PLEs in the whole population and in two separate groups. Considering that demographic variables, such as age, might correlate both with wisdom and PLEs, we also tested the interaction between demographic variables and wisdom in the association between wisdom and frequency/distress of PLEs in each group using moderation models. Finally, we conducted moderation analysis to explore the moderating effect of wisdom on the relationship between the frequency of PLEs and associated distress in the whole group or in each of the two groups alone after controlling the demographic variables. PROCESS for Windows was used to build a moderation model (43). Bootstrap inference with 5,000 samples and heteroskedasticity-consistent standard error estimator (HC4) were adopted considering the violation of normal distribution, which was in accordance with our previous study (44). IBM SPSS 24.0 for Windows (45) was used for other statistical analyses.

## Results

### Sample Characteristics

Our online survey recruited 927 participants from thirteen Chinese provinces in total. We eliminated: 35 subjects for their previous diagnosis of any psychiatric illness, 37 subjects for spending less than 3 min on this survey, and 4 subjects for apparent mismatch between reported age and education years. No participant was discarded for missing value because the online survey can only be submitted with every question completed. After cleaning the data, a total of 851 participants entered the final data analysis, including 7.6% middle school students, 69.5% undergraduate students, 4.8% graduate students, 13.3% employed staff, and 4.8% the retired. Other details of sample characteristics are shown in Table 1.

**TABLE 1:** Sample characteristics.

*N* = 851	
**Demographics**	
Females, n (%)	591 (69.4)
Age, years, mean (S.D.)	23.7 (8.5)
Han ethnicity, n (%)	805 (94.6)
Education, years, mean (S.D.)	14.3 (2.8)
Family history, n (%)	20 (2.4)
**Assessments, mean (S.D.)**	
3D-WS-12	39.4 (5.6)
Cognitive	12.4 (2.5)
Affective	14.0 (2.3)
Reflective	13.0 (2.3)
CAPE-P15, frequency, total	20.1 (4.2)
Persecutory ideation	7.7 (1.9)
Bizarre experiences	9.1 (2.2)
Perceptual abnormalities	3.4 (0.9)
CAPE-P15, distress, total	8.1 (7.2)
Persecutory ideation	4.5 (3.4)
Bizarre experiences	3.1 (3.6)
Perceptual abnormalities	0.5 (1.4)

*3D-WS-12 = 12-Item Abbreviated Three-Dimensional Wisdom Scale; CAPE-P15 = 15-Item positive subscale of Community Assessment of Psychic Experiences; S.D. = Standard Deviation.*

### Group Comparison Between Participants With High-Level and Low-Level Psychotic-Like Experiences

Using the cut-off value of 1.47 in the mean frequency score of CAPE-P15 (41, 42), we identified 188 individuals with high-level PLEs (PLEs group, 22.1%) and 663 individuals with low-level PLEs (low-PLEs group, 77.9%). Three-dimensional wisdom total score was lower in the high-PLEs group, along with the cognitive, affective and reflective components (Table 2).

**TABLE 2:** Difference between high-PLEs and low-PLEs group in demographics and assessments.

	High-PLEs (*n* = 188)	Low-PLEs (*n* = 663)	Statistics	*p* value	Cohen’s d
**Demographics**					
Females, n (%)	116 (61.7)	475 (71.6)	6.4	0.012	\
Age, years, mean (S.D.)	21.6 (7.5)	24.2 (8.6)	32.8	<0.001	0.31
Han ethnicity, n (%)	176 (93.6)	629 (94.9)	0.4	0.503	\
Education years, mean (S.D.)	13.0 (2.7)	14.7 (2.7)	7.0	<0.001	0.62
Family history, n (%)	4 (2.7)	16 (2.3)	4.1	0.388	\
**Assessments, mean (S.D.)**					
3D-WS-12, total	36.5 (5.4)	40.2 (5.4)	59.9	<0.001	0.68
Cognitive	11.7 (2.5)	12.6 (2.5)	18.0	<0.001	0.35
Affective	13.0 (2.4)	14.3 (2.1)	46.8	<0.001	0.59
Reflective	11.8 (2.2)	13.3 (2.2)	59.3	<0.001	0.69
CAPE-P15, frequency, total	26.5 (3.6)	18.6 (2.0)	443.2	<0.001	3.29
Persecutory ideation	10.0 (1.8)	7.0 (1.3)	315.8	<0.001	2.08
Bizarre experiences	12.2 (2.3)	8.2 (1.2)	366.3	<0.001	2.68
Perceptual abnormalities	4.3 (1.4)	3.1 (0.4)	258.2	<0.001	1.59
CAPE-P15, distress, total	18.5 (6.8)	5.2 (3.7)	397.5	<0.001	2.93
Persecutory ideation	8.6 (3.0)	3.3 (2.5)	289.9	<0.001	1.99
Bizarre experiences	8.0 (4.1)	1.7 (1.8)	336.8	<0.001	2.50
Perceptual abnormalities	2.0 (2.3)	0.1 (0.6)	258.6	<0.001	1.51

### Associations Between Wisdom and Psychotic-Like Experiences

At the population level, wisdom was negatively correlated with total frequency and associated distress of PLEs, which was replicated in three subtypes of PLEs: persecutory ideation (PI), bizarre experiences (BE), and perceptual abnormalities (PA). These results were verified in each dimension of wisdom (cognitive, affective, and reflective) (Table 3).

**TABLE 3:** Spearman’s correlations between wisdom and other variables in the whole population.

	3D-WS-12 Cognitive	3D-WS-12 Affective	3D-WS-12 Reflective	3D-WS-12Total
**Demographics**				
Gender (higher = males)	−0.03	−0.04	0.02	−0.02
Age	0.08*	0.09**	0.19***	0.15***
Education	0.09**	0.09**	0.15***	0.14***
**Assessments**				
CAPE-P15, frequency, total	−0.20***	−0.31***	−0.36***	−0.35***
Persecutory ideation	−0.20***	−0.28***	−0.37***	−0.34***
Bizarre experiences	−0.17***	−0.28***	−0.29**	−0.30***
Perceptual abnormalities	−0.08*	−0.14***	−0.15***	−0.15***
CAPE-P15, distress, total	−0.22***	−0.31***	−0.39***	−0.37***
Persecutory ideation	−0.23***	−0.28***	−0.39***	−0.36***
Bizarre experiences	−0.18***	−0.28***	−0.32***	−0.31**
Perceptual abnormalities	−0.08*	−0.15***	−0.15***	−0.15***

*p < 0.05, **p < 0.01, ***p < 0.001; 3D-WS-12 = 12-Item Abbreviated Three-Dimensional Wisdom Scale; CAPE-P15 = 15-Item positive subscale of Community Assessment of Psychic Experiences.

In the high-PLEs group, correlations between wisdom and frequency/distress of PLEs were still significant at the total score level, but wisdom was not correlated with the frequency and distress of perceptual abnormalities (PA), nor in any of the three wisdom subscales. It should be noted that reflective wisdom showed a slightly strong negative correlation with the total frequency and distress score of PLEs compared to cognitive and affective wisdom (Table 4).

**TABLE 4:** Spearman’s correlations between wisdom and other variables in the high-PLEs group.

	3D-WS-12 Cognitive	3D-WS-12 Affective	3D-WS-12 Reflective	3D-WS-12Total
**Demographics**				
Gender (higher = males)	−0.11	−0.03	−0.11	−0.11
Age	0.06	−0.03	0.10	0.05
Education	0.01	−0.08	0.01	0.01
**Assessments**				
CAPE-P15, frequency, total	−0.11	−0.18*	−0.22**	−0.21**
Persecutory ideation	−0.20**	−0.11	−0.23**	−0.23**
Bizarre experiences	−0.08	−0.19*	−0.22**	−0.22**
Perceptual abnormalities	−0.01	−0.01	−0.01	0.01
CAPE-P15, distress, total	−0.18*	−0.21**	−0.24***	−0.28***
Persecutory ideation	−0.27***	−0.10	−0.26***	−0.28***
Bizarre experiences	−0.10	−0.20**	−0.21**	−0.25**
Perceptual abnormalities	−0.02	−0.07	−0.04	−0.03

*p < 0.05, **p < 0.01, ***p < 0.001; 3D-WS-12 = 12-Item Abbreviated Three-Dimensional Wisdom Scale; CAPE-P15 = 15-Item positive subscale of Community Assessment of Psychic Experiences.

In the low-PLEs group, the total three-dimensional wisdom score was correlated with the total frequency and distress score of PLEs and PI/BE subscales except PA, as seen in the high-PLEs group. Other results were similar to findings in the high-PLEs group and the whole population (Table 5). Further analysis revealed that no significant interaction between demographic variables (age, gender, education) and total wisdom score was observed in the association between wisdom and frequency/distress of PLEs.

**TABLE 5:** Spearman’s correlations between wisdom and other variables in the low-PLEs group.

	3D-WS-12 Cognitive	3D-WS-12 Affective	3D-WS-12 Reflective	3D-WS-12Total
**Demographics**				
Gender (higher = males)	−0.02	−0.08	0.02	−0.03
Age	0.05	0.06	0.16***	0.11**
Education	0.07	0.06	0.13**	0.11**
**Assessments**				
CAPE-P15, frequency, total	−0.15***	−0.22***	−0.28***	−0.28***
Persecutory ideation	−0.14***	−0.18***	−0.29***	−0.25***
Bizarre experiences	−0.11**	−0.18***	−0.17***	−0.18***
Perceptual abnormalities	0.01	−0.02	−0.03	−0.02
CAPE-P15, distress, total	−0.17***	−0.18***	−0.32***	−0.28***
Persecutory ideation	−0.17***	−0.18***	−0.32***	−0.27***
Bizarre experiences	−0.13***	−0.17***	−0.21***	−0.20***
Perceptual abnormalities	0.01	−0.02	−0.03	−0.02

**p < 0.01, ***p < 0.001; 3D-WS-12 = 12-Item Abbreviated Three-Dimensional Wisdom Scale; CAPE-P15 = 15-Item positive subscale of Community Assessment of Psychic Experiences.

### Moderation of Wisdom on the Relation Between Psychotic-Like Experiences Frequency and Their Associated Distress

**FIGURE 1:**
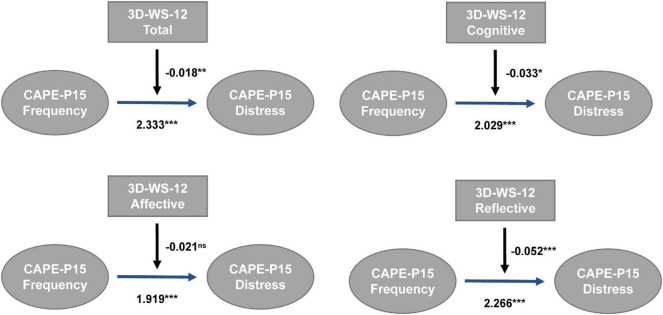
Moderation of wisdom in the relation between PLEs frequency and associated distress in low-PLEs group (*N* = 663, PROCESS Model 1, ns, not significant, *p < 0.05, **p < 0.01, ***p < 0.001).

At the whole population level, after controlling for age, sex, and educational years, we observed no significant moderating effect of wisdom total score on the relation between PLEs frequency and associated distress, nor that of any of the three subscales of wisdom score. This was replicated in the high-PLEs group. However, in the low-PLEs group, total wisdom score significantly attenuated the relation between total PLEs frequency and associated distress [coefficient = −0.018, Bootstrap 95% CI (−0.029, −0.008)]. At the subscales level, this moderation existed in the cognitive [coefficient = −0.033, Bootstrap 95% CI (−0.058, −0.008)] and reflective [coefficient = −0.052, Bootstrap 95% CI (−0.079, −0.024)] wisdom but not in affective wisdom (Figure 1). We further tested this moderation on the subscales of CAPE-P15 (PI, BE, and PA), but the moderation of wisdom was only significant at the total frequency and distress level of PLEs.

## Discussion

To the best of our knowledge, this is the first work to investigate the association between wisdom and subclinical psychotic symptoms, and has three major findings. First, the three-dimensional wisdom level was decreased in participants with high-level PLEs compared to those with less PLEs. Second, wisdom was negatively associated with frequency and associated distress of PLEs. Third, wisdom attenuated the relation between the frequency of PLEs and their associated distress, but only when the frequency was relatively low.

To date, three-dimensional wisdom has not been measured in the subclinical psychotic population, but a previous study suggested that schizophrenia patients had lower wisdom scores (including cognitive, affective, and reflective wisdom) than the control group (46); our findings of decreased wisdom in the subclinical psychotic population highly resonate with these findings. Studies investigating the wisdom level as a whole in the subclinical psychotic population are still lacking. However, some aspects of wisdom were covered in previous studies. A large body of research concluded that many forms of cognitive biases were observed in individuals with PLEs, such as jumping to conclusions (JTC), aberrant salience (ASB), attention to threat (ATB), externalizing bias (ETB), and belief inflexibility (BIB) (30, 47–50). Notably, it is also well-established that patients with psychosis presented cognitive dysfunction that could precede the onset of full-blown disease with debilitating positive symptoms and cognitive distortion even contribute to the maintenance of psychotic symptoms (51). This suggests that the clinical and subclinical psychotic population might share similar alterations in cognitive function but with varying degrees. Cognitive wisdom, however, refers to an individual’s ability to understand life, to comprehend the significance and deeper meaning of phenomena and events, especially with regard to intrapersonal and interpersonal matters, such as the knowledge of the positive and negative aspects of human nature (6). Decreased cognitive wisdom in the population with high-level PLEs is likely to explain the existence of cognitive biases or distortions, which can possibly translate to cognitive symptoms of psychotic patients.

Notably, our results highlight the critical role of reflective wisdom in terms of association with common mental health domains covered in this study. Reflective wisdom is considered to be a prerequisite for developing cognitive wisdom. Perception of reality without any major distortions is mandatory to reach a deeper understanding of life. Wise individuals would engage in reflective thinking by looking at phenomena and events from different perspectives to develop self-awareness and self-insight (3, 6). Previous studies found that individuals with PLEs are prone to poor reflective reasoning and biased self-reflection (52, 53). In clinical settings, a large portion of patients with full-blown psychosis suffer from impaired insight and thus are difficult to reach a rational understanding of their conditions (54, 55). Our work provided further evidence that individuals with more PLEs showed undermined reflective capability, which can be explained by decreased reflective wisdom.

Affective wisdom represents positive emotions and behavior toward others, such as sympathy and compassion. Dysfunction in empathy can be observed in individuals at high risk for psychosis (56). At clinical settings, patients at risk for psychosis showed significant impairment in social cognition, specifically including difficulties in emotion recognition and social skills (36). Further, dysfunction in emotion regulation was associated with the frequency and distress of PLEs (37). In line with these findings, our results provided evidence that individuals with PLEs were in lack of affective wisdom, which is critical in forming social bonds with others.

Furthermore, individuals with higher wisdom tended to have less frequent PLEs and lower degrees of associated distress in the PLEs group. Notably, these findings also apply to the low-PLEs group, suggesting a general protective effect of wisdom for mental health. In these associations, reflective wisdom seemed to play a more significant role among the three wisdom dimensions, which was also observed in a wisdom study on schizophrenia patients (46) and can possibly be explained by the prerequisite role of reflective wisdom for generating cognitive and affective wisdom (6). For the three domains of PLEs, compared to persecutory ideation and bizarre experiences, it should be noted that wisdom showed little ameliorating effect on perceptual abnormalities, which implies that subclinical hallucinations and delusions might share a different nature.

Notably, the moderation results revealed that wiser individuals tended to have lower distress even with the same frequency of PLEs. However, this moderation was only significant among the participants who reported lower levels of PLEs (the low-PLEs group). It could be interpreted that wisdom can attenuate the distress associated with PLEs when the frequency is relatively low. When the frequency reaches a specific threshold, this moderation would no longer work due to the general deterioration of the individual’s condition and the possible loss of insight. Further, this moderation seemed more stronger for reflective wisdom, in accordance with its critical role in three-dimensional wisdom as aforementioned. Our findings were also supported by previous works. Cognitive biases moderated the relation between negative affective states and PLEs in non-clinical adults (57). Individuals having difficulties in emotion regulation showed more frequent PLEs and distress (37).

Our findings may have important implications for clinical practices. Wisdom is considered to be amenable to intervention (58) and thus can be trained and cultivated, just like resilience (59). Under this notion, tailored intervention targeting three dimensions of wisdom can potentially ameliorate the frequency and associated distress of PLEs in this non-clinical population, and meanwhile contribute to a better general mental health outcome. For instance, it could be hypothesized that enhancement of cognitive wisdom could prevent cognitive biases, reflective wisdom could improve the self-reflection, and affective wisdom may boost one’s empathy and compassion and finally improve social cognition and function. Works examining these promising hypotheses are highly recommended to lower the debilitating burden of full-onset psychosis to the individual family and to society as a whole.

Finally, our results should be interpreted with several limitations. First, the cross-sectional design limited the conclusion of causal relationship, and further cohort studies following the outcomes of individuals with different levels of wisdom is recommended. Second, the convenience sampling method may incur bias and have less generalizability. For instance, we recruited more females possibly because women are more likely to seek mental health services and participate in psychiatric studies (60, 61). Thus, works using probability sampling (62) to target more representative population are warranted. Third, the sample size of participants with high-level PLEs are relatively modest, future replication in larger population is also needed. Lastly, wisdom may be taken in a different manner since a previous work failed to replicate the three-dimension structure of 3D-WS-12 (63), thus, the interpretation of our results should be cautious, and further validation of three-dimensional wisdom structure is warranted to get a better understanding of its utility in Chinese culture.

Together, our work investigated the association between wisdom and PLEs for the first time, finding that wisdom ameliorated the frequency/distress of PLEs, and attenuated the distress associated with PLEs. Our findings have good implications for clinical practice since wisdom serves as a protective role for PLEs and could be a promising target in preventing the transition from high-risk state to full-blown psychosis.

## Data Availability Statement

The raw data supporting the conclusions of this article will be made available by the authors, without undue reservation.

## Ethics Statement

The studies involving human participants were reviewed and approved by the Ethics Committee of The Second Xiangya Hospital of Central South University. Written informed consent to participate in this study was provided by the participants’ legal guardian/next of kin.

## Author Contributions

ZL, YL, and ZWu designed the study. ZJ, FW, ZWa, and YJ collected the data and double-checked the data input. ZWu and XS finished the statistical analysis and drafted the manuscript. ZL, YL, ZJ, and BR revised the manuscript. All authors agreed on the final version of the manuscript.

## Conflict of Interest

The authors declare that the research was conducted in the absence of any commercial or financial relationships that could be construed as a potential conflict of interest.

## Publisher’s Note

All claims expressed in this article are solely those of the authors and do not necessarily represent those of their affiliated organizations, or those of the publisher, the editors and the reviewers. Any product that may be evaluated in this article, or claim that may be made by its manufacturer, is not guaranteed or endorsed by the publisher.

## Footnotes

1
www.wjx.cn
